# A one health approach to vaccines against *Toxoplasma gondii*

**DOI:** 10.1016/j.fawpar.2019.e00053

**Published:** 2019-04-18

**Authors:** Elisabeth A. Innes, Clare Hamilton, Joao L. Garcia, Andreas Chryssafidis, David Smith

**Affiliations:** a Moredun Research Institute, Pentlands Science Park, Edinburgh, Scotland EH26 OPZ, United Kingdom of Great Britain and Northern Ireland; b Universidade Estadual de Londrina, Campus Universitario, Rodovia Celso Garcia Cid, Pr 380, CEP 86057-970 Londrina, Parana, Brazil; c Department of Veterinary Medicine, Universidade do Estado de Santa Catarina, Lages, SC, Brazil; d 5740A Medical Science Building II, 1150 W. Medical Centre Dr, University of Michigan, Ann Arbor, MI 48109-5620, USA

**Keywords:** *Toxoplasma gondii*, Vaccines, One health, Animals, Humans

## Abstract

Toxoplasmosis is a serious disease with global impact, now recognised as one of the most important food borne diseases worldwide and a major cause of production loss in livestock. A one health approach to develop a vaccination programme to tackle toxoplasmosis is an attractive and realistic prospect. Knowledge of disease epidemiology, parasite transmission routes and main risk groups has helped to target key host species and outcomes for a vaccine programme and these would be to prevent/reduce congenital disease in women and sheep; prevent/reduce *T. gondii* tissue cysts in food animal species and to prevent/reduce *T. gondii* oocyst shedding in cats. Most animals, including humans, develop good protective immunity following infection, involving cell mediated immune responses, which may explain why live vaccines are generally more effective to protect against *T. gondii*. Recent advances in our knowledge of parasite genetics and gene manipulation, strain variation, key antigenic epitopes, delivery systems and induction of immune responses are all contributing to the prospects of developing new vaccines which may be more widely applicable. A key area in progressing vaccine development is to devise standard vaccine efficacy models in relevant animal hosts and this is where a one health approach bringing together researchers across different disciplines can be of major benefit. The tools and technologies are in place to make a real impact in tackling toxoplasmosis using vaccination and it just requires a collective will to make it happen.

## Highlights

• A one health integrated vaccine programme recommended for animals and people
• Vaccine targets to reduce: oocyst shedding, cyst development and congenital disease
• Live vaccines are: effective, induce protective immunity, have safety issues
• Killed vaccines are desirable and recent scientific advances increase feasibility
• Vaccine efficacy testing: appropriate host, standardised challenge model

## 1 Introduction

In 2008, a very significant conference was held in Buzios, near Rio de Janeiro in Brazil to celebrate 100 years since the discovery of *Toxoplasma gondii*. The real impact of this Toxoplasma Centennial Congress was to bring together researchers from many different disciplines including those involved in medicine, public health, veterinary research, food safety, biology, genetics, environmental and water science, all studying different aspects of the same parasite and all together in one plenary session for the whole conference. It was very appropriate that the conference was held in Brazil as Splendore working in Brazil (Splendore, 1908) along with Nicolle and Manceaux in North Africa (Nicolle and Manceaux, 1908), were the first to reference the discovery of *T. gondii* as a new species in the scientific literature. There is also some very interesting research arising from Brazil looking at the disease incidence and severity along with increased genetic diversity of *T. gondii* that is very different from the situation in Europe and North America (Dubey et al., 2012). Having key scientists researching *T. gondii* from across the world all in one place at the conference in Buzios sharing their knowledge and most importantly getting the perspectives of their colleagues in very different disciplines really helped to encourage “One Health” thinking and discussions on how to tackle *T. gondii* using a more inter-disciplinary and collaborative approach.

*Toxoplasma gondii* lends itself to a one health approach to tackle the diseases it causes as there is only one species that occurs worldwide. The parasite can infect nearly all warm blooded animals, and we have a solid understanding of the parasite genome, lifecycle, transmission routes and disease epidemiology. Additionally, the parasite is readily maintained and manipulated in the laboratory. Indeed, it is this ease of manipulation which has led *T. gondii* to become a model organism for studying many aspects of parasite biology (Boothroyd, 2009). So why has there not been more progress in developing tools to tackle toxoplasmosis globally and what would be the advantages in taking a one health approach to do this?

Vaccination is an approach that could greatly reduce the transmission and disease impact of *T. gondii*. In general, following infection, most animal species including people develop good protective immune responses to the parasite that will usually protect against disease going forward (Innes et al., 2009). However, despite the significant numbers of research papers looking at vaccination against *T. gondii* over the last 30 years, there is currently only one vaccine available worldwide which is licenced for use in sheep to protect against abortion due to *T. gondii* infection (Buxton and Innes, 1995). The reasons for this may be that the parasite was prioritised by farmers and veterinarians as being of great concern to the sheep industry worldwide, being one of the most important causes of infectious abortion in sheep and goats (Buxton and Rodger, 2008). There was an economic and welfare need to develop a vaccine and it is generally easier to get regulatory approval for veterinary vaccines compared to those being used in humans.

To date, progress on the development of a vaccine for use in humans has been slow which may be because it is generally not considered a priority for public health. Some new research looking at measuring impact of disease has now highlighted *T. gondii* as one of the most important food borne pathogens in Europe and USA (Batz et al., 2012; Torgerson and Mastroiacovo, 2013; Havelaar et al., 2015). Using a measure called disability adjusted life years (DALYS) the impact of disease caused by *T. gondii* can be quantified (Havelaar et al., 2015). This has highlighted the impact of the disease in congenitally infected children who suffer brain damage, eye and auditory disease for life. As there are no drugs or treatments available to cure persistent *T. gondii* infection, efforts should be made to develop a vaccine to prevent human infection and reduce disease.

The other area for a vaccination approach is to use our knowledge of the main transmission routes of *T. gondii* to people and look at the strategic use of vaccination in other key animal hosts to improve public health, through reduction of tissue cysts in food animals and reduce environmental contamination through vaccination of cats to reduce shedding of oocysts.

In this review we will look at the main risk groups for disease and where intervention using vaccination might be applied with reference to knowledge of the life cycle of the parasite, the main transmission routes and disease epidemiology. In discussing vaccine development we will review progress in understanding key protective immune responses, live and killed vaccine approaches, relevant model systems and progress in vaccine development in key host species. Finally, we will look at the advantages in adopting a one health approach that employs vaccination to reduce *T. gondii* infections and disease in humans and animals.

## 2 Risk groups and impact of disease

Infection with *T. gondii* can cause a wide range of clinical consequences in people and animals (Innes, 1997). Most people who become infected with *T. gondii* have very minor symptoms and would often be unaware they had come into contact with the parasite. However, if a pregnant woman has a primary infection with the parasite the consequences can be very severe for the developing foetus resulting in brain damage, eye and/or auditory disease, and sometimes even death depending on the stage of pregnancy when infection occurs (Wallon and Peyron, 2018). The incidence of congenital infection in France, where they conduct a national screening programme is estimated to be 2:10000 births (Villena et al., 2010), in Brazil it is estimated to be 1:1000 births (Dubey et al., 2012) and in the USA 1:10000 births (McAuley, 2014) and due to the severity of the long term consequences for the affected child, the impact of the disease is significant. Some recent epidemiology studies using DALYS as a measure of disease impact ranked *T. gondii* as one of the most important food borne pathogens worldwide (Torgerson and Mastroiacovo, 2013).

Immuno-compromised individuals are also an important risk group for infection with *T. gondii* as they are unable to effectively control parasite multiplication. Patients with acquired immune deficiency syndrome (AIDS) that have persistent *T. gondii* infection, may present with severe brain lesions where the *T. gondii* parasites within tissue cysts become active again and start multiplying due to the dysfunction of their cell-mediated immune system which would otherwise keep the parasite in check (Luft et al., 1984). People undergoing immune suppressive treatments, such as with cancer chemotherapy or organ transplant, may also be at risk from *T. gondii* infection (Wang et al., 2017).

There are also reports of severe acute toxoplasmosis in immunocompetent individuals occurring in French Guiana (Carme et al., 2002) and cases of ocular disease in immunocompetent individuals in Brazil (Dubey et al., 2012).

The high prevalence of *T. gondii* in the human population and the wide range of different diseases attributed to infection with the parasite make *T. gondii* a highly significant global pathogen with a likely underestimated impact.

The parasite is also a significant cause of congenital disease in sheep and goats worldwide (Buxton and Rodger, 2008). Veterinary surveillance statistics in the UK over the last 5 years from 2014 to 2018 has found that *T. gondii* is the cause of 28% of ovine abortions (APHA Veterinary Gateway Livestock disease surveillance dashboards www.apha.gov.uk). The annual incidence rates of clinical toxoplasmosis in sheep in the UK have been estimated to be between 1 and 2% per annum (Blewett and Trees, 1987) which if extrapolated to Europe, having approximately 66 million breeding ewes, the potential losses of lambs due to toxoplasmosis would be around 1,320,000. Therefore, the parasite has a significant economic and welfare impact for livestock farmers globally.

It is thought that evolution and genetics may play a role in susceptibility to disease and animals that have largely evolved away from the cat and have not been exposed to oocysts are very vulnerable to infection with *T. gondii.* Examples of these are marsupials, new world monkeys and marine mammals who are very vulnerable to infection and can suffer acute fatal disease (Dubey, 2010).

## 3 *T. gondii* life cycle and main transmission routes

Toxoplasma is one of the most successful parasites worldwide, capable of infecting all warm blooded animals and it is estimated that around 20–25% of the human population are infected. Felids are the only definitive host and the only host species where the sexual life cycle of the parasite can occur resulting in the shedding of *T. gondii* oocysts in faeces (Dubey, 2010). Young cats usually become infected for the first time when hunting and eating birds or rodents infected with *T. gondii*. Tissue cysts containing *T. gondii* bradyzoites are ingested by the cat and the digestive enzymes in the gut break down the cysts wall releasing bradyzoites that invade the epithelial cells in the intestine enabling the parasite to develop within the host cells. The sexual cycle of *T. gondii* occurs in the gut of the cat with the female macrogamonts and the male microgamonts being found throughout the small intestine, most commonly in the ileum. The microgametes fertilise the macrogametes resulting in zygote formation and an oocyst wall is formed around the parasite (Dubey, 2010). The epithelial cells containing the oocysts rupture and release the oocysts into the gut of the cat where they are excreted in the faeces. The oocysts are not infectious for another host until they have sporulated and this process occurs within a few days outside the cat in the environment. Each oocyst comprises two sporocysts each containing four sporozoites. The oocysts have a very tough outer shell and are able to survive for long periods of time in the environment, particularly in temperate and moist environments (Lelu et al., 2012). Sporulated oocysts are infectious for new hosts if they are consumed either by grazing animals or through consumption of contaminated food or water (Dubey, 2010). An infected cat may shed millions of oocysts over a week or two following a primary infection with *T. gondii* and then they develop immunity to the parasite which in general will prevent them shedding oocysts going forward (Dubey, 1995). The cat population is very widespread across the world with approximately one third of households in the USA (Dubey, 2010) and 26% of households in the UK (Murray et al., 2010) owning a cat. Following a primary infection with *T. gondii* cats may shed millions of oocysts into the environment and it only requires 1–10 oocysts to infect a mouse (Dubey, 2006; Muller et al., 2017), 100 oocysts to infect another cat (Dubey, 2006) and 200 oocysts to cause infection in a naïve sheep (McColgan et al., 1988). Infection is much more efficient in cats via the ingestion of tissue cysts, (Dubey, 2006) and this is likely to be an evolutionary adaptation and demonstrates the suitability of the *T. gondii* lifecycle to carnivorism in cats (Dubey, 2010).

As cats are a critical host for *T. gondii* and the only host animal species capable of shedding the environmentally resistant and long lived oocyst stage of the parasite, the cat is a key target host species for a one health vaccination approach to tackle *T. gondii*.

In other animal or human hosts, transmission of *T. gondii* may occur through consumption of oocysts in contaminated food or water or through eating raw or undercooked meat from other animals persistently infected with *T. gondii* and harbouring tissue cysts containing bradyzoites (Dubey, 2010). Following consumption of oocysts by intermediate hosts, sporozoites excyst and invade the enterocytes and cells of the ileal epithelium and convert to tachyzoites, the rapidly multiplying stage of the parasite (Dubey, 2010). The tachyzoites can penetrate most nucleated host cells forming a parasitophorous vacuole and multiply using a process called endodyogeny. The parasite will keep dividing within host cells until the host cell ruptures releasing the tachyzoites that will go on and invade and multiply within other host cells. A key stage in the parasite life cycle within the host is the conversion from tachyzoite to bradyzoite (slow multiplying stage) which enables the parasite to “hide” from the immune system of the host within an infected cell where there is a modification of the parasitophorous vacuole to form a cyst wall (Ferguson and Hutchison, 1987). The bradyzoites multiply very slowly within these tissue cysts and in the majority of host species are thought to persist for the lifetime of the host where they can be found in brain, eyes, heart and skeletal muscles (Dubey, 2010). In response to parasite invasion and multiplication in host cells the host's immune system becomes activated and is thought to play an important role in triggering stage conversion from tachyzoite to bradyzoite as a means of the parasite to evade the immune response and remain hidden within the host (Frenkel, 2000).

Consumption of *T. gondii* bradyzoites within tissue cysts in raw or undercooked meat is a common transmission route for carnivorous hosts and the tissue cyst wall is broken down by the digestive enzymes in the stomach and intestines releasing the bradyzoites which penetrate and multiply within the intestinal cells. The parasite then continues its life cycle in the new host with the fast multiplying tachyzoite stage and then differentiation into the slow multiplying bradyzoite stage within tissue cysts. Epidemiology studies have emphasised the importance of the consumption of raw or undercooked meat containing *T. gondii* bradyzoites as a major transmission route for people (Cook et al., 2000).

A key target for a one health vaccination programme would be to vaccinate food livestock to prevent or reduce development of *T. gondii* tissue cysts in the meat.

If a primary infection occurs during pregnancy the tachyzoite stage of the parasite can be carried via the circulation to the placenta where the parasite can invade and infect the cells of the placenta and then spread to the developing foetus where the parasite can cause severe disease (Remington et al., 1995). Congenital toxoplasmosis can also occur in sheep and goats where a primary infection with *T. gondii* can lead to abortion and neonatal mortality (Buxton and Rodger, 2008). In pregnant sheep, *T. gondii* tachyzoites invade and multiply within the maternal tissues of the placentome where they can then transmit to the trophoblast cells of the foetus (Buxton and Finlayson, 1986). The stage of gestation when infection occurs is critical to the outcome, with infection occurring early in pregnancy having the most severe consequences for the ovine foetus. Infection occurring later in pregnancy when the foetal immune system is better developed, will have less severe clinical consequences (Innes et al., 2009). This is very similar to the situation with pregnant women where the foetus will suffer more severe clinical effects if *T. gondii* infection occurs early in pregnancy compared to later in gestation (Jacquemard, 2000).

A key target for a one health vaccination programme would be to prevent congenital disease in pregnant women and susceptible animal hosts such as sheep or goats.

## 4 Host immunity in the context of vaccine development

Following the initial invasion of host tissues by *T. gondii,* the host's immune system is stimulated to respond to the infection and in most cases the immune system is able to contain it. The response generated limits the acute phase of the infection and restricts the persistent phase. The importance of the immune system in containing the infection is demonstrated by reactivation of the acute phase associated with immunosuppression, observed in cases of Toxoplasma-associated encephalitis in immune-compromised AIDS patients (Luft et al., 1984). After a first infection with *T. gondii*, it is possible for the host to become protected against subsequent exposure to the parasite suggesting that vaccination is a feasible approach to help prevent and control disease (Innes et al., 2011). The key to developing a safer, more efficacious vaccine against toxoplasmosis in both humans and livestock will be dependent on our understanding of the protective host immune response during *T. gondii* invasion and infection and how this can be recapitulated without a live parasite.

A key component of protective immunity involves the cytokine Interferon gamma (IFN-γ) which is involved in both innate and adaptive immunity (Suzuki et al., 1988; Tait and Hunter, 2009; Yarovinsky, 2014; Sasai et al., 2018). Production of this cytokine results in the regulation of antimicrobial responses, including inducible nitrous oxide synthase (iNOS) expression, the production of nitric oxide (NO) and reactive oxygen species (ROS), and intracellular tryptophan restriction, all of which serve to inhibit *T. gondii* multiplication in the host (Nathan et al., 1983; Pfefferkorn, 1984). In order to generate a protective IFN-γ response, activated macrophages, dendritic cells (DCs) and neutrophils must be stimulated to produce interleukin-12 (IL-12). During early infection, IL-12 from activated myeloid cells stimulates the proliferation of natural killer (NK) cells as part of an innate immune response (Gazzinelli et al., 1993; Gazzinelli et al., 1994; Hunter et al., 1994; Yarovinsky, 2014). Long-term protection against *T. gondii* requires the activation of CD4+ and CD8+ T cells (Sasai et al., 2018). Together, NK cells, CD4+ and CD8+ T cells produce large quantities of IFN-γ to facilitate a protective T_h_1 immune response against acute and reactivated toxoplasmosis (Sasai et al., 2018). Activation of these immune cells also promotes an antibody response to help control acute toxoplasmosis (Filisetti and Candolfi, 2004; Correa et al., 2007). Dendritic cells and macrophages also play a role as antigen-presenting cells that recognise *T. gondii*-infected cells and stimulate the recruitment of monocytes and neutrophils via the up-regulation of the chemokines CCL2 and CXCL2 (Sasai et al., 2018). Monocytes and neutrophils are then stimulated to express IFN-γ. In the brain, IFN-γ-recruited CD8+ T cells have been shown to eliminate latent cysts via the secretion of the cytolytic protein perforin (Persson et al., 2007; Suzuki et al., 2011). This information demonstrates which immune cell types must be stimulated in order to generate protection against *T. gondii* infection. A successful vaccine will be particularly dependent on the timely production of IFN-γ and the associated outcomes of IFN-γ production by these cells.

Design of effective vaccines requires an understanding of the key protective immune responses and much of our knowledge of these comes from experimental studies in mice. However, it is important to keep in mind that while much can be learned from using mouse models of *T. gondii* infection it is not always appropriate to directly extrapolate research from murine immune responses to other host species.

Early in infection of the host, *T. gondii* secretes profilin (TgPF), an actin-remodelling protein essential for cell invasion and egress (but not intracellular growth). In mouse models of infection, TgPF represents a major parasite agonist and an important pathogen-associated molecular pattern (PAMP) containing unique epitopes that are recognised by Toll-like receptors 11 (TLR11) and 12 (TLR12) on conventional dendritic cells (DCs) and macrophages (specifically TLR12 on plasmacytoid DCs) (Yarovinsky et al., 2005; Plattner et al., 2008; Koblansky et al., 2013). Recognition by TLR11 and/or TLR12 leads to the up-regulation of IL-12, which in turn triggers the production of IFN-γ in mice (De Sousa et al., 1997; Yarovinsky et al., 2005). Vaccination of C57BL/6 mice with recombinant TgPF elicits an IFN-γ response and, following challenge with the parasite, lowers cyst burden in the brain and improves survival (Tanaka et al., 2014). However, exposure of human myeloid cells to TgPF failed to generate an IFN-γ response (Tombácz et al., 2018). In part, this could be due to the human genome lacking functional genes for encoding TLR11 and TLR12 and having no orthologous counterpart for recognising TgPF as a major PAMP (Roach et al., 2005; Gazzinelli et al., 2014). Similarly, while vaccination of mice with *T. gondii* surface antigens is sufficient to elicit protection (Uggla et al., 1988; Øvernes et al., 1991; Buxton and Innes, 1995), a comparable vaccine tested in sheep failed to protect pregnant ewes following challenge with the parasite (Buxton and Innes, 1995).

It has been shown that human monocytes and DCs exposed to TgPF-deficient parasites can still produce the pro-inflammatory cytokines IL-12 and tumour necrosis factor alpha (TNF-α) (Tosh et al., 2016; Sher et al., 2017). In humans, recognition of *T. gondii* by myeloid cells and subsequent IL-12 up-regulation requires the phagocytosis of live parasites, as it has been shown that phagocytosis of heat-killed tachyzoites does not produce a cytokine response (Subauste and Wessendarp, 2000; Sher et al., 2017). A similar response has been observed in sheep, whereby prior inoculation with inactive tachyzoites failed to protect pregnant ewes against a subsequent challenge with *T. gondii* (Beverley et al., 1971; Wilkins et al., 1987; Buxton and Innes, 1995). Therefore, a phagocytosis event may be required in cattle, sheep and pigs for generating a protective immune response, since the TLR11 and TLR12 receptors important in mediating an early immune response in mice are also not present in the bovine, ovine or porcine genomes (Uenishi and Shinkai, 2009).

Invasion of a cell by the parasite prevents a protective response, partly due to the resistance of the parasitophorous vacuole (PV) to fusion with host cell lysosomes (Tosh et al., 2016; Sher et al., 2017). Phagocytosed parasites, on the other hand, will fuse with the lysosome to form a phagolysosome. Subsequently, parasites will be degraded and a resulting IL-12 response induced (Sher et al., 2017). A protective immune response is only achieved when live parasites are phagocytosed perhaps due to the timely secretion of parasite proteins during the phagocytosis event. This would support the concept of new approaches to vaccine development based on genetically modified live parasites that are incapable of invading a host cell but are still susceptible to phagocytosis. This would differ from vaccination with killed parasites, as vaccination with live parasites would allow for the secretion of parasite proteins during phagocytosis that may be important to the generation of a protective immune response.

## 5 A one health approach to vaccination

A vaccine is a material originating from a pathogen that induces an immunologically mediated resistance to disease (Mims, 1982) and is an approach that has great potential to be effective against *T. gondii*. Most animals (including humans) develop a robust immune response following infection which affords protection against disease going forward and the same species of parasite occurs across the world although there is genetic and biological diversity of different *T. gondii* strains. The parasite is easy to manipulate and culture in the laboratory, there have been extensive studies to understand host immune responses in various animal species and information on the *T. gondii* genome is now available (www.toxodb.org). To date, there is only one commercially available vaccine which is licenced for use in sheep to help protect against *T. gondii* associated abortion (Innes et al., 2011). Despite the high impact of human disease globally, there are currently no human vaccines. Using information on disease epidemiology, transmission routes and primary risk groups, a one health vaccination strategy could be developed to reduce the impact of *T. gondii* in both people and animals (see Fig. A). The key objectives of a vaccine programme would be to: prevent congenital disease in both women and vulnerable animal species such as sheep and goats through preventing parasites from invading and establishing infection in the placenta; prevent/reduce *T. gondii* tissue cysts in food animals; and prevent/reduce oocyst shedding in cats. The outcomes of the vaccination approaches are different and involve protection against congenital disease, production of safe meat for human consumption and reducing environmental contamination with infective oocysts. These different approaches and outcomes illustrate the advantages of adopting a one health strategy to develop a vaccination programme targeting both humans and animals to achieve maximum impact in tackling toxoplasmosis. In the following sections we will review current knowledge in developing vaccines in these key target areas.

**Fig. A:**
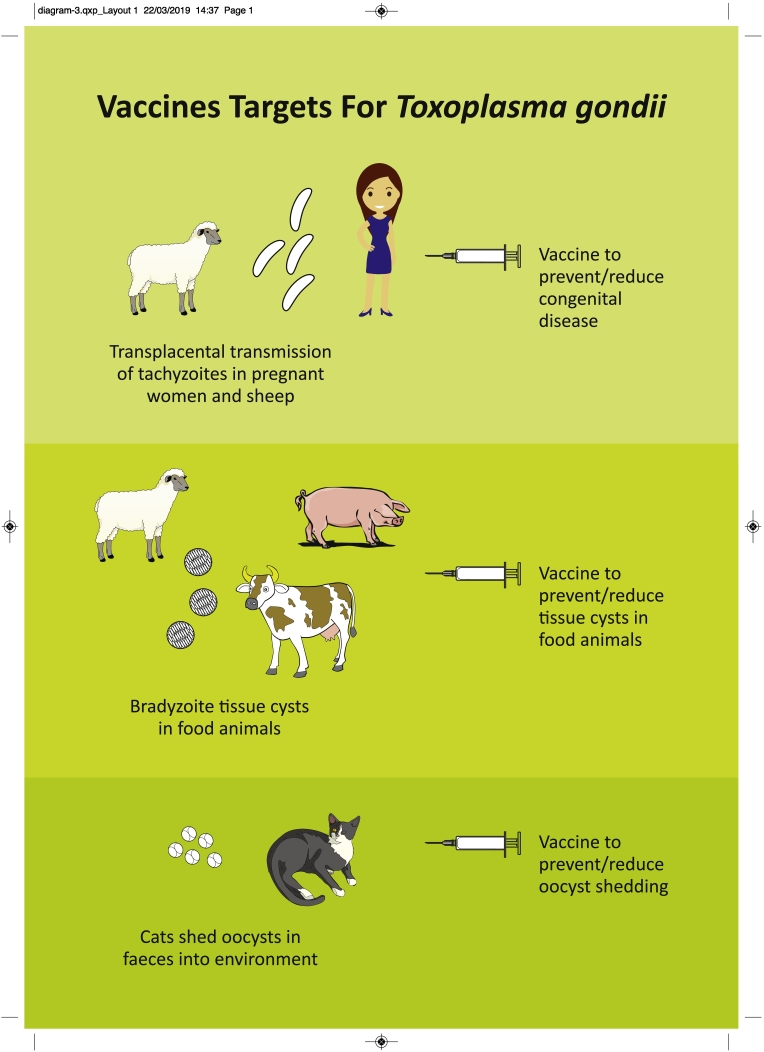
Vaccine targets for a one health approach to tackle toxoplasmosis

### 5.1 Vaccines to prevent congenital toxoplasmosis

#### 5.1.1 Human vaccine

A major target for a human vaccine would be to prevent the severe consequences of congenital toxoplasmosis in pregnant women. *T. gondii* infection will generally only be a risk to the developing foetus if a pregnant woman becomes infected for the first time during pregnancy. Women who have experienced infection with the parasite prior to pregnancy generally develop effective immunity to protect them against transplacental transmission in subsequent pregnancies (Henriquez et al., 2010). A vaccination programme may be best achieved through targeting adolescent girls similar to the current programme of vaccination to protect against human papillomavirus (HPV), (Levy-Bruhl et al., 2009). The reasons for this would be to try and induce protective immunity to *T. gondii* in young women prior to them becoming pregnant and therefore reducing the risk of *T. gondii* infection during pregnancy. While live vaccine preparations are likely to be more effective in stimulating appropriate protective cell-mediated immune responses to *T. gondii* there are safety and regulatory issues that may impede the use of live vaccines in humans (Innes et al., 2011). Killed vaccines would be a safer option and although there are considerable challenges to overcome, recent scientific advances lend some optimism for the prospect of an effective human vaccine. New information is now available on the genomes of the main *T. gondii* strains (www.toxodb.org) and knowledge of the key protective immune responses involving MHC class I restricted CD8+ T-cells and IFNу may help to determine predictive aligorithms for protective epitopes (Henriquez et al., 2010; Cong et al., 2011). In addition, the immunosense approach has identified immunogenic peptides that were able to elicit IFNу responses in peripheral blood lymphocytes from *T. gondii* seropositive humans but not from *T. gondii* negative humans and were found to elicit protective immunity when used to immunise human MHC I (HLA) transgenic mice (Cong et al., 2010; Bissati et al., 2016). With new advances in our understanding of the impact of *T. gondii* on human health and information on the key protective immune responses there is increasing interest in developing a vaccine for use in people. Key challenges will be to induce appropriate protective immune responses using non-viable parasites and having a suitable efficacy model to test a human vaccine. As congenital disease caused by *T. gondii* is similar in women and sheep, an efficacy model using sheep may be helpful in progressing a vaccine to prevent congenital disease in women (Innes and Vermeulen, 2006).

#### 5.1.2 Sheep vaccine

Sheep can become infected with *T. gondii* through the consumption of sporulated oocysts contaminating pasture, feed and water and if a primary infection occurs when the ewe is pregnant the tachyzoite stage of the parasite can invade and multiply within the placenta and infect the developing foetus resulting in abortion or birth of a still born lamb (Buxton and Rodger, 2008). Sheep develop good protective immunity following a primary infection which will prevent transplacental transmission following subsequent infectious challenge (Innes et al., 2009). Studies involving the characterisation of local in vivo immune responses in sheep during a primary infection with *T. gondii* were done using cannulation of the efferent duct of a peripheral lymph node close to the site of challenge (Innes et al., 1995). The key immune responses induced during a primary infection of sheep involved initially IFNγ and activated CD4+ T-cells, then activated CD8+ T cells followed by detection of specific antibodies (Innes and Wastling, 1995).

Research in New Zealand found that a strain of *T. gondii,* designated S48, which had originally been isolated from an aborted lamb, had significantly changed as a result of being passaged in mice for many years and was no longer able to differentiate into bradyzoites and tissue cysts (Buxton, 1993). This S48 “incomplete” strain was able to infect and multiply within host cells and therefore would stimulate protective host cell mediated immune responses, but would not persist within the animal. The strain was initially tested in New Zealand as a vaccine to see if it would stimulate immunologically mediated protection in ewes against congenital toxoplasmosis and it was found to be very effective (O'Connell et al. (1988)). The S48 vaccine underwent further evaluation in experimental trials in Scotland where naïve ewes were vaccinated prior to mating and then challenged at mid gestation. The vaccinated ewes were significantly protected against a challenge that caused abortion in naïve control animals (Buxton and Innes, 1995). A further group of vaccinated ewes were kept for 18 months in the absence of any *T. gondii* challenge and then given a further experimental oral challenge with oocysts during pregnancy and were found to be solidly immune (Buxton et al., 1993) showing that the immunological protection induced following vaccination is long lived in the absence of further challenge.

In the field situation, the vaccine is advised to be used in ewes at least 3 weeks prior to mating and one shot should give long term protection as the ewe will also get a boost to this immunity through picking up the infection naturally when grazing. The vaccine is live and has a short shelf life of a few weeks so it is very important that it is administered within the time specified as it will not be effective if the tachyzoites are no longer viable. The S48 strain live vaccine (Toxovax ™) is the only commercial vaccine worldwide to protect against congenital toxoplasmosis in sheep and is available in UK, Ireland, France and New Zealand through MSD. The S48 vaccine is highly effective because it is a live vaccine and because it will invade host cells and undergo limited multiplication and the *T. gondii* antigens are presented to the host immune system in the correct manner to enable induction of protective immune responses (Innes et al., 2011). The main drawback of this vaccine is that it is live which may give rise to safety concerns and it has a short shelf life which can lead to issues in production and distribution (Innes et al., 2011).

The efficacy of a gene knockout strain of *T. gondii* (RH strain) lacking the mic 1 and mic 3 genes (mic 1–3 KO) was tested for its ability to induce protective immunity against *T. gondii* induced abortion in sheep (Mevelec et al., 2010). The deletion of the mic 1 and 3 genes in RH strain reduces the virulence of RH in mice. Vaccination of naïve ewes with the MIC 1–3 KO *T. gondii* parasites 2 months prior to mating was found to protect against an oocyst challenge in mid-gestation that caused abortion in non-vaccinated control animals (Mevelec et al., 2010). The MIC 1–3 KO parasites were found to persist as tissue cysts in the brains of the vaccinated sheep.

A killed vaccine would be very desirable but the challenge with a killed vaccine is that they are generally less effective in stimulating the required protective cell mediated immune responses (Innes et al., 2011). Previously reported studies using killed whole tachyzoite preparations as vaccines did not protect sheep against subsequent challenge with *T. gondii* (Beverley et al., 1971 and Wilkins et al., 1987). *T. gondii* membrane antigens formulated into immune stimulating complexes (ISCOMS) were tested for their ability to induce protective immune responses in sheep. While the vaccine preparation did modify the effects of the experimental challenge, with lower lamb mortality observed compared to the un-vaccinated control group, the results were not found to be significant (Buxton et al., 1989).

A crude extract of *T. gondii* tachyzoites encapsulated into poly(lactide-*co*-glycolide) micro and nano particles were used to immunise sheep intranasally to try and stimulate a protective mucosal immune response (Stanley et al., 2004). The immunisation was able to stimulate both humoral and cell mediated immunity along with mucosal IgA responses although no protection against challenge with oocysts was observed (Stanley et al., 2004). The potential of DNA vaccines has also been evaluated in sheep involving four different dense granule antigens from *T. gondii* (GRA1, 4, 6 and 7) where it was shown that an intramuscular administration route resulted in stimulation of specific antibody responses and induction of IFNу (Hiszcynska-Sawicka et al., 2011). This study only looked at induction of immune responses and did not look at any challenge infections to test efficacy of protective immunity.

The impetus to develop a vaccine to protect against congenital toxoplasmosis in sheep is clear due to the significant economic and welfare impacts the disease has on the sheep livestock industry across the world. The other significant factor is that vaccination studies can be done in a relevant animal model, the sheep, which greatly helps in assessing the robustness, relevance and safety of data when making decisions about licensing vaccines for commercial use. The sheep models to study *T. gondii* infection in pregnant and non-pregnant animals have been well established (Buxton and Innes, 1995; Innes et al., 2009) with detailed information available on disease pathogenesis, dose, strain, timing and route of inoculations which are of great value in testing new vaccine preparations going forward. The sheep may also be a more relevant animal model than the mouse to test potential human vaccines to protect against congenital toxoplasmosis as the disease in pregnant sheep and pregnant women has similarities (Innes and Vermeulen, 2006).

### 5.2 Vaccines to prevent/reduce tissue cysts in food animals

Epidemiology studies have emphasised the importance of consumption of raw or undercooked meat from *T. gondii* infected food animals as a major transmission route for human infection (Slifko et al., 2000; Cook et al., 2000; EFSA, 2007). Recent studies have examined prevalence of *T. gondii* in the main food animal species in Europe (Opsteegh et al., 2016) where the parasite can be found in sheep, pigs (in particular outdoor reared pigs (Kijlstra et al., 2004), horses, chicken and cattle. A recent opinion from the European Food Safety Authority suggests that meat-borne transmission accounts for around 60% of *T. gondii* infections with the major contributing sources being *T. gondii*-infected beef, pork and small ruminant meat (EFSA, 2018). The strategic application of vaccination of food animals was suggested as a means of preventing/reducing viable tissue cysts in meat making it safer for human and animal consumption (EFSA, 2018); however, to make this feasible, more work needs to be done on identifying the relative risk of infection from the different livestock groups.

Cattle are reported to be clinically resistant to *T. gondii*, clearing infection to undetectable levels within a few weeks (Burrells et al., 2018; Dubey, 1983), and as such are often thought to pose less risk to consumers. However, a study conducted in the Netherlands involving a quantitative risk assessment for meat-borne toxoplasmosis reported that although the incidence of *T. gondii* was much lower in cattle than in outdoor reared pigs, beef contributed to the majority of predicted human cases due to the higher likelihood of it being consumed raw or undercooked compared to pork (Opsteegh et al., 2011a, Opsteegh et al., 2011b). To our knowledge there have been no vaccination studies on naturally or experimentally infected cows to determine if there is a reduction in tissue cyst formation. Given the discrepancy between indirect and direct detection of *T. gondii* in cows (Burrells et al., 2018; Opsteegh et al., 2011a, Opsteegh et al., 2011b), more sensitive methods would be required to be able to assess vaccine efficacy in these animals.

Meat from *T. gondii* infected pigs has been highlighted as a concern for human infection due to large quantities of pig meat consumed worldwide. The risk of acquiring *T. gondii* from pork will depend on the region of the world and the husbandry system used with outdoor reared animals being a higher risk for infection with the parasite than indoor reared (Kijlstra et al., 2004). A study in the United States estimated that 41% of foodborne toxoplasmosis cases were attributable to the consumption of pork, making it a major source of infection (Batz et al., 2012).

Pigs are infected with *T. gondii* mainly via the ingestion of water, food and soil contaminated with sporulated oocysts as well as ingestion of animals harbouring *T. gondii* tissue cysts. Because of this, the control of cats and animal vectors, such as birds and rodents, are critical for toxoplasmosis control in pig herds (Mateus-Pinilla et al., 1999; Suijkerbuijk et al., 2018).

There is currently no commercial vaccine available for use in pigs (Garcia, 2009; Innes et al., 2009); however, some studies with live *T. gondii* vaccine candidates have been carried out in pigs using the TS-4, S48 and RH strains, which will undergo multiplication in the host and help to stimulate protective immune responses (Dubey et al., 1991). Vaccination of pigs with the RH strain of *T. gondii* found that the vaccine did not persist in pig tissues after 64 days and that the vaccinated pigs were protected from the development of *T. gondii* tissue cysts following oral challenge with oocysts (Dubey et al., 1991). The addition of oligodeoxynucleotides containing immunostimulatory CpG motifs as an adjuvant was found to enhance the immune responses in pigs immunised with live RH strain tachyzoites (Kringel et al., 2004). Immunisation of pigs with tachyzoites of the TS-4 temperature sensitive mutant strain of *T. gondii*, showed that the TS-4 tachyzoites did not persist in the pigs following vaccination but the immunity induced did not completely protect against tissue cysts development following challenge with *T. gondii* oocysts (Pinckney et al., 1994).The live S48 *T. gondii* strain, an incomplete strain that has lost the ability to differentiate into bradyzoites and is used as a vaccine to protect against congenital toxoplasmosis in sheep, was used to immunise pigs and was found to offer significant protection against the development of tissue cysts following an oral challenge with *T. gondii* oocysts (Burrells et al., 2015).

Ingestion of raw or undercooked lamb and goat meat is also a potentially significant risk factor for infection with *T. gondii*. In two studies in the United States, 27.1% of 383 lambs sampled at slaughter and 53.4% of 234 goat hearts purchased from a retail store were seropositive for *T. gondii* (Dubey et al., 2008; Dubey et al., 2011). Furthermore, viable parasites could be isolated from 51% of seropositive lamb hearts and 26% of seropositive goat hearts bioassayed in mice. A study with the S48 vaccine strain of *T. gondii* has shown that it will also protect sheep against the development of tissue cysts following an oral challenge with *T. gondii* oocysts, highlighting the potential double benefit for farmers vaccinating their sheep flocks (Katzer et al., 2014). Although the commercial vaccine is licenced for use in goats to prevent abortion, to our knowledge it has not been tested to determine whether a there is a reduction in tissue cyst formation similar to that seen in sheep.

The live vaccines tested so far in pigs have been found to be very effective in inducing immunity to protect against the development of tissue cysts following further challenge, however there are safety concerns with the use of such vaccines and also some issues with large scale production and distribution along with a short shelf life. There have also been studies to look at the efficacy of killed vaccines which should be safer than live vaccines but have challenges of being able to induce protective cell mediated immune responses (Jongert et al., 2009).

The potential for killed vaccines has been assessed in pigs. A killed vaccine using a lysate of *T. gondii* tachyzoite antigens incorporated into immunostimulating complexes ISCOM as an adjuvant was found to induce good antibody responses after three immunisations (Freire et al., 2003). The ISCOM vaccination regime was found to protect pigs against tissue cyst development following an oral challenge with *T. gondii* oocysts (Freire et al., 2003). Two further studies evaluated crude *T. gondii* rhoptry proteins to vaccinate pigs using either an ISCOM adjuvant with subcutaneous administration (Garcia et al., 2005) or Quil A with intranasal administration (da Cunha et al., 2012) and both the studies reported partial protection against tissue cyst development following oral challenge with *T. gondii* oocysts. The ability of a GRA1-GRA7 cocktail DNA vaccine was tested in pigs following intradermal inoculation and was found to induce strong humoral and cell mediated immune responses including the key cytokine IFN-y which is known to be important in protective immunity against *T. gondii* (Jongert et al., 2008). A further vaccine trial using a subcutaneous administration of excreted and secreted antigens of *T. gondii* was conducted in pigs which resulted in a reduction in the development of tissue cysts following a live challenge compared to the controls groups (Wang et al., 2013).

Therefore, vaccination of food animal species is an option to produce safer meat for human consumption with live vaccines showing the greatest efficacy. A one health approach would help to drive this forward as the impetus to vaccinate the livestock would be for a public health as opposed to a veterinary health benefit.

### 5.3 Vaccines to prevent/reduce oocyst shedding in cats

Cats play a key role in toxoplasmosis transmission and epidemiology, as they are the only species that can shed environmentally resistant oocysts that can cause infection and disease in many other host species including humans (Carruthers, 2002; de Moura et al., 2006; Almeida et al., 2011; Torrey and Yolken, 2013; Chiang et al., 2014; Cornelissen et al., 2014; Schlüter et al., 2014).

Cats shed oocysts for a short period after an initial infection and develop an effective immune response, which usually protects the animals against shedding further oocysts due to a new infection (Dubey and Frenkel, 1974; Davis and Dubey, 1995). Exceptions to this general rule have been reported where occasionally immunocompetent cats can re-shed oocysts, especially when re-infected with a heterologous strain (Dubey, 1995; Zulpo et al., 2018), and immunocompromised cats may re-shed oocysts even without a second infection (Dubey and Frenkel, 1974).

Based on the immunological memory generated by a first exposure, vaccination of cats may be an effective strategy for reducing oocyst burden in the environment (Hermentin and Aspöck, 1988; Dubey, 1996; Torrey and Yolken, 2013; Zhang et al., 2013; Opsteegh et al., 2015).

Some experiments suggested that antigenic differences between parasite strains may result in strain specific immunity, with protection detected only when the immunising and challenge strains were the same (Dubey and Frenkel, 1974; Omata et al., 1996). Live ^60^Co-irradiated tachyzoites of *T. gondii* Beverley strain were inoculated subcutaneously in cats, generating protection against oocyst shedding in 43% of immunised cats, but only when the animals were infected with the homologous strain (Omata et al., 1996). When the immunised cats were challenged with a heterologous strain (RH strain), no protection was detected and all cats shed oocysts following challenge. Cats fed ME49 strain tissue cysts eliminated oocysts, but 89% of them did not re-shed when inoculated with heterologous strains, suggesting that the enteric parasite cycle can also have some role in the development of protective immunity (Freyre et al., 2007).

A mutant *T. gondii* strain that is unable to produce oocysts in cats, called T-263 strain, was isolated and employed for cat immunisation (Frenkel et al., 1991). Kittens fed once with T-263 live tissue cysts did not produce oocysts and, despite the absence of parasite multiplication and consequent mucosal priming of the immune response, the infection prevented 84% of the kittens from passing oocysts after challenge with a complete (heterologous) parasite strain (Frenkel et al., 1991). On the other hand, kittens fed two doses of T-263 tissue cysts, demonstrated complete protection against a further challenge, with no animal shedding oocysts (Freyre et al., 1993). The same immunity level was achieved using two oral doses of free bradyzoites, released from cysts after artificial digestion, but no protection against oocysts shedding was elicited when live T-263 tachyzoites were used for immunisation (Freyre et al., 1993). The T-263 bradyzoite vaccine was evaluated in a long-term field trial on swine farms (Mateus-Pinilla et al., 1999). Oral vaccination using T-263 bradyzoites of the majority of farm cats was able to significantly reduce *Toxoplasma* infection in finishing pigs (Mateus-Pinilla et al., 1999). As a result, cat vaccination, together with cat population control and environmental cleaning to reduce oocyst survival, would all contribute to the control of *T. gondii* in finishing pigs, consequently lowering the number of tissue cysts in pork and hence minimizing the risk of human exposure through consumption of undercooked infected pork (Mateus-Pinilla et al., 2002).

In spite of the positive results and effectiveness of T-263 bradyzoite vaccine for immunising cats, there are some issues relating to the large scale production and distribution of the vaccine as it is composed of live organisms. Bradyzoites of the T-263 strain must be recovered from persistently infected mice, limiting large scale production. The vaccine would need to be transported frozen, in liquid nitrogen, and be thawed no more than 15 min before application and the vaccine could accidently infect the operator as it comprises live parasites (Choromanski et al., 1995). As a result the T-263 vaccine has not been taken forward yet for commercial production.

Second generation vaccines produced with subunit antigens of the parasite are desirable to overcome those issues related to the use of live vaccines. The inoculation of feline herpesvirus type 1 (FHV1) genetically modified to express a *T. gondii* rhoptry protein (Rop2) induced humoral immune responses and decreased parasite development in challenged cats, but the FHV-ROP2 vaccine did not prevent oocyst shedding in any animal (Mishima et al., 2002).

Crude rhoptry proteins were used in a vaccination trial with cats (Garcia et al., 2007). Three vaccine doses were delivered through the intranasal route, to help stimulate the mucosal immune response. A reduction of 67% in oocyst shedding was found in the immunised group, and two of three animals did not shed any oocysts after challenge (Garcia et al., 2007). Different routes of administration of this same crude rhoptry protein vaccine, with an additional dose, were evaluated (Zulpo et al., 2012). Four intranasal doses promoted a reduction of 98% in oocyst shedding, while four intra-rectal doses reduced the shedding by 53%; however, neither vaccine produced complete protection in the tested animals, as all cats passed some parasites in the faeces (Zulpo et al., 2012). In a similar trial, cats received four intranasal doses of a recombinant Rop2 protein (rROP2) vaccine which resulted in a reduction of 87% in oocyst elimination, but animals that received bovine serum albumin also presented 80% less oocysts than unvaccinated and challenged animals (Zulpo et al., 2017).

Clearly a vaccine that can prevent or reduce oocyst shedding in cats would be an important component of a one health vaccination programme and it would be interesting to model how effective such a vaccine would have to be to make a difference to environmental contamination with oocysts and hence reduce the risk of infecting further intermediate hosts. A mathematical modelling approach suggested that the application of a continuous vaccination programme in cats to prevent/reduce oocysts shedding would be an effective control strategy to tackle toxoplasmosis (Arenas et al., 2010). Rodent control was not included in these mathematical analyses, but can be very important for preventing feline infection and controlling parasite spread (Jiang et al., 2012).

An important consideration in testing new vaccines is to use a standardized vaccine challenge model (Szabo and Finney, 2017) as it can be very difficult to compare the efficacy of different candidate vaccines due to differences in the strains of *T. gondii* used, the life cycle stages, the route and dose of inoculation which all have a profound effect on the outcome of the challenge. A standardized challenge model was proposed to test the efficacy of novel vaccines in cats (Cornelissen et al., 2014) and it would certainly be helpful for researchers working in this area to adopt a standard protocol for testing vaccines which would enable valid comparisons to be made on efficacy, reduce the numbers of animals used in research and hopefully speed up the process of vaccine development through collaboration.

A further thought in developing a vaccine for use in cats would be a strategy to persuade cat owners to pay for and use such a vaccine to protect public health as opposed to the health of their cat. A study looking at the influencing factors in creating a vaccine programme for *T. gondii* in cats highlighted the importance of cost of the vaccine to the successful uptake (Sykes and Rychtář, 2015).

## 6 Genetic diversity and relevance to vaccine design

The population structure of *T. gondii* was originally thought to be clonal, with most isolates belonging to one of three lineages, designated Type I, Type II and Type III (Howe and Sibley, 1995). While the study which defined these lineages was extremely important, the samples included in the analysis were primarily from clinical cases from Europe and North America and, therefore, not representative of the global *T. gondii* population. Since then, it has become apparent that unlike Europe and North America where the clonal lineages predominate, in South and Central America these clonal lineages are rare and there is an overwhelming abundance of non-clonal (atypical) genotypes (Shwab et al., 2014). Similarly, in Africa and Asia, the clonal lineages exist alongside other locally common genotypes (Chaichan et al., 2017; Galal et al., 2018). Following comprehensive studies assessing strain abundance, geographical distribution and genetic diversity, the *T. gondii* population is now known to consist of 15 haplogroups clustered into six major clades (Khan et al., 2007; Su et al., 2012). Along with the clonal lineages (Types I, II and III) other main genotypes have been identified, including BrI, BrII, BrIII and BrIV in South and Central America, Type 12 in North America, Africa 1 in Africa and Chinese 1 in Asia (Chen et al., 2011; Khan et al., 2011; Mercier et al., 2010; Pena et al., 2008).

In a recent study, the genotypes of over 1400 *T. gondii* isolates originating from every continent except Antarctica (no samples) and Australia (too few samples) were compared and a total of 189 genotypes were identified (Shwab et al., 2014). Although the number of isolates from each continent varied, the difference between the number of genotypes identified from North America, Europe, Asia and Africa in comparison to South and Central America was stark. In North America and Europe, over 75% of isolates were Type II, III or Type 12, in Asia almost 50% of isolates were Chinese 1 and in Africa almost 85% were Type II (variant) or Type III. In complete contrast, 156 genotypes were identified in South and Central America with no one genotype clearly dominating.

A number of explanations have been proposed to explain the difference in genetic diversity between the northern hemisphere and South America. Given the diversity in South America, it is thought that *T. gondii* originated in this region and only a small number of founding strains were introduced to North America and other continents via maritime trading of agricultural goods (possibly contaminated with soil containing oocysts) and accidental transportation of cats and rodents (Lehmann et al., 2006). These founding populations could then expand with limited recombination giving rise to the clonal structure observed in the northern hemisphere. It has also been suggested that the wider range of definitive hosts present in South America in comparison to North America and Europe, as well as the tropical climate has allowed for greater oocyst survival resulting in more infections and the possibility of mixed infections and recombination giving rise to a more diverse population (Shwab et al., 2014).

### 6.1 Virulence of *T*. *gondii* strains

Virulence of *T. gondii* has been extensively studied in the mouse model and can vary depending on a number of factors, including strain of mouse used and genotype of the infecting parasite. The degree of virulence of the clonal lineages has been well defined, with Type I isolates causing 100% mortality irrespective of the inoculating dose and Types II and III causing intermediate or no mortality, depending on dose (Robert-Gangneux and Darde, 2012). Virulence is usually measured by inoculating outbred mice with either the digested tissues of an infected animal/patient (bioassay) or a defined dose of tachyzoites or oocysts and then recording the numbers of mice requiring euthanasia due to clinical toxoplasmosis. An isolate is classed as virulent if it causes 100% mortality in the mice, as moderately virulent if it causes 30–99% mortality and as avirulent if it causes less than 30% mortality (Pena et al., 2008). However, given that there is no control over infecting dose in a bioassay and that the strain of mice, infecting stage of the parasite and the route of inoculation can all influence the outcome of infection, it is extremely difficult to make meaningful comparisons between studies. Recently, Saraf et al. (2017) highlighted the issues around the determination of virulence and put forward a standardized methodology which would facilitate more productive comparisons in future studies. This would involve testing a series of concentrations of tachyzoites in outbred mice, monitoring numbers of euthanasias, testing serum of survivors using the modified agglutination test (MAT) and then determining the cumulative mortality (Saraf et al., 2017).

Determining virulence in human cases is more difficult due to the limited number of samples from clinical cases but overall it appears that strains containing Type I or atypical alleles are more pathogenic or more likely to cause severe disease than other isolates. Severe disseminated toxoplasmosis has been described in healthy, immune competent people in South America (French Guiana and Suriname) and in Europe (France) infected with atypical strains of the parasite (Carme et al., 2009; Demar et al., 2007; Pomares et al., 2011). The high pathogenicity of atypical strains in South America is thought to be responsible for the increased incidence of severe ocular toxoplasmosis reported in Brazil where the diagnostic prevalence is 18% compared to 2% in Europe and the USA (Glasner et al., 1992; Holland, 2003). Also, congenitally infected children in Brazil are five times more likely to develop severe ocular lesions leading to visual impairment than European children (Gilbert et al., 2008). In a study on congenital toxoplasmosis (CT) in the USA, prematurity and severe disease at birth were significantly associated with a non-Type II serotype (McLeod et al., 2012). Clonal Type II and Type III genotypes have also been associated with severe CT, particularly in France where Type II strains dominate; however, the outcome of infection is clearly linked to the point of gestation when the mother becomes infected (Ajzenberg et al., 2002). Severe CT is more common when maternal infection with a Type II strain occurs in early pregnancy and is rare when infection occurs in the third trimester; whereas severe CT can result with an atypical strain irrespective of the point of gestation when infection occurs (Ajzenberg et al., 2002; Delhaes et al., 2010; Xiao and Yolken, 2015). Furthermore, cases of CT due to atypical strains have poorer outcomes regardless of treatment (Delhaes et al., 2010). It is also possible for previously immune people and animals to become re-infected with an atypical strain resulting in severe CT or abortion storms, respectively, highlighting the greater pathogenicity of these strains and the lack of cross protective immunity (Edwards and Dubey, 2013; Elbez-Rubinstein et al., 2009).

### 6.2 Strain-specific immunity and virulence factors

The immune response to clonal strains of *T. gondii* has been well characterised in the mouse model. Early production of IL-12 by macrophages and the subsequent production of interferon (IFN)-у by stimulated natural killer cells, CD4^+^ and CD8^+^ T cells are key to parasite control (Dupont et al., 2012). Strain-dependent immune responses have been described. In response to infection with Type II strains, there is increased production of IL-12 and other pro-inflammatory cytokines, recruitment of immunity-regulated GTPases (IRGs) which act to destroy the parasitophorous vacuole, production of nitric oxide and IFN-у, recruitment of CD8^+^ T cells and ultimate control of the parasite (reviewed in Melo et al. (2011). A similar response has been documented in response to infection with a Type III strain, although the pro-inflammatory response is not as pronounced likely explaining why this strain, unlike Type II, is almost exclusively avirulent (Sibley and Boothroyd, 1992). In contrast to Types II and III, infection with a Type I strain results in decreased production of IL-12 (and other pro-inflammatory cytokines) and no recruitment of IRGs to the parasitophorous vacuole allowing the parasite to replicate uncontrollably causing death due to excessive parasite burden (Melo et al., 2011).

In a study of South American and European patients with active ocular toxoplasmosis (OT), levels of IFN-у and IL-17 were significantly higher in the vitreous humour from French patients compared to the Columbian patients where they were barely detectable (de-la-Torre et al., 2013). Furthermore, genotyping revealed that Columbian patients were infected with type I and atypical strains whereas the French patients were infected with Type II strains. Given the known protective effects of IFN-у, the suppressed levels seen in Columbian patients could explain the increased incidence of severe OT in South America.

Polymorphic rhoptry (ROP) proteins are secreted by the parasite upon invasion and have been shown to play a role in host-parasite interactions and immune evasion (Hunter and Sibley, 2012). In a recent study of 240 *T. gondii* strains from South America and Asia, the allelic profile of ROP proteins was correlated with virulence data and ROP5 and ROP18 were identified as key determinants of virulence in mice (Shwab et al., 2016). These proteins have been shown to inhibit IFN-у-induced recruitment of IRGs thus aiding survival of the parasite (Niedelman et al., 2012). It is important to note, however, that humans lack the same number of IRG genes as mice so while ROP proteins are likely to play some role in determining virulence, it may also involve other parasite and host genetic factors.

Although it has been recognised for many years that previous infection with *T. gondii* can protect against subsequent infection, it has now become apparent that re-infection is possible in both animal and human hosts, which may have potential implications for vaccination. However, in most incidences where re-infection is reported, the secondary infecting strain is distinct from the primary challenge strain. For example, mice infected with a clonal strain are protected from challenge with a clonal strain but not atypical strains (Elbez-Rubinstein et al., 2009; Jensen et al., 2015). Similarly, in a case of severe CT diagnosed in France, where Type II strains dominate, the strain isolated from the baby was an atypical genotype not found in Europe (Elbez-Rubinstein et al., 2009). Studies in mice have also shown that primary challenge with an atypical strain protects against infection with a challenging virulent atypical strain (Costa et al., 2018). Given that the clonal lineages are 98–99% similar, it is possible to attain heterologous immunity due to common immune-dominant antigens; however, it is unclear if this immunity would extend to atypical strains and may pose a challenge for future vaccine design.

## 7 New approaches

Understanding protein function is important in the development of novel control strategies against *T. gondii* and other infectious organisms. One approach to uncovering the biological roles of parasite proteins and determining their importance is to utilize compounds that block their function. However, various factors can limit this approach, including the specificity of the compound in question and its toxicity to the host. Another method to understanding protein function can be to generate a recombinant version of the protein, however this approach also has limitations. Recombinant proteins do not always express as desired, with technical issues arising with regards to yield, purity and folding conformation (Gräslund et al., 2008; Young et al., 2012). Furthermore, experiments can only be performed ex situ, that is, in a user-defined context outside of the parasite, which can limit interpretations with regards to parasite biology.

A new and highly effective approach to uncovering protein function is to genetically modify parasites. A recent review highlights the emergence of CRISPR/Cas9 technology in apicomplexan parasites, including *T. gondii*, and demonstrates its effectiveness in furthering understanding of gene function and parasite biology (Di Cristina and Carruthers, 2018). This approach allows endogenous *T. gondii* genes to be tagged, which in turn allows the corresponding protein to be tracked in the parasite during host cell infection (Shen et al., 2014). Tagged proteins can also be purified from parasite lysates for study outside of the parasite (Braun et al., 2008; Huynh and Carruthers, 2009). Another application of CRISPR/Cas9 technology is to modify genes so that the translated amino acid sequence is altered. This can be performed to truncate proteins, to remove specific domains, or to modify specific codons and analyse the contribution of specific amino acids with regards to protein function (Guerra et al., 2018). Furthermore, this approach can be used to remove entire genes from the parasite's genome. One of the most impressive studies to utilize CRISPR/Cas9 technology in this way was a genome-wide knockout of each *T. gondii* gene (Sidik et al., 2016; Sidik et al., 2018). In this work, the authors were able to individually interrogate the role and importance of every *T. gondii* gene during the parasite lytic cycle, in turn providing a “phenotype score” for each gene (Sidik et al., 2016). Establishing which proteins are essential to specific aspects of parasite biology and the host protective response is relevant for the identification of novel vaccine targets.

The permanent disruption of a gene essential to the lytic cycle prevents parasites from being maintained in a cell culture system, which can limit how much information can be gained on a particular gene. One way around this is to develop an inducible knockdown strain. Many studies have employed the use of inducible knockdown to study the roles of specific and essential *T. gondii* genes. An example of this is the tetracycline repressor-based inducible system. Parasites are genetically modified such that transcription of the gene-of-interest is under the control of the tetracycline-regulated transactivator system (Meissner et al., 2001). When parasites are cultured in the presence of anhydrotetracycline (ATc), expression of the target gene is down-regulated. Direct responses to specific gene knockdown can subsequently be made. Alternatively, a TetON system could be implemented in which expression of the gene-of-interest is only sustained in the presence of ATc (Etheridge et al., 2014). Initially limited to conditional gene expression during the acute stage, ATc has recently been shown to cross the cyst wall of bradyzoites (Krishnamurthy and Saeij, 2018), thus this approach can be utilised for studying gene function across the different parasite life cycle stages.

In the context of this review it is relevant to consider the effectiveness of the TetON system in the development of a novel live vaccine for *T. gondii*. During laboratory cell culture, parasites could be maintained in the presence of ATc to ensure the expression of a gene essential for completion of the lytic cycle (e.g. host cell invasion). However, once parasites are taken out of this environment and used in a vaccine challenge experiment in vivo, expression of the essential gene would be down-regulated. This would prevent parasite invasion and dissemination in the host, allowing phagocytosis of live parasites to take place and a protective immune response to be stimulated. Alternatively, genes essential to bradyzoite conversion could also be targeted with this approach, preventing the development of a persistent infection following in vivo challenge. This would be similar to the S48 strain (Toxovax™) that is used to vaccinate sheep (Buxton and Innes, 1995). Since the genome of the S48 strain has not been characterised to-date, the mutations responsible for its attenuation have yet to be unravelled. Doing so would certainly be insightful for the targeted genetic manipulation of *T. gondii*, in the pursuit of a live-attenuated vaccine strain with greater utility in a one health vaccination programme.

Another approach that has recently been implemented in *T. gondii* for studying the effects of conditional protein knockdown has been the auxin-inducible degradation (AID) system. A gene-of-interest is tagged with an AID domain (or miniAID domain, mAID) that becomes ubiquitinated by the TIR1-SCF E3 ubiquitin kinase complex in the presence of auxin (indole-3-acetic acid, IAA). Ubiquitination results in the protein being degraded via the proteasome (Brown et al., 2018). Although requiring the use of *T. gondii* strains modified to express the auxin receptor TIR1, this system is highly efficient, with protein degradation occurring in as little as fifteen minutes (Brown et al., 2017). Furthermore, the AID-system can be applied in cell culture experiments and in murine models of infection, due to the non-toxic properties of IAA (Brown and Sibley, 2018). Demonstrating the effectiveness of this approach for conditional protein knockdown, researchers have utilised the AID system to interrogate the roles of calcium-dependent protein kinase 1 (CDPK1), two cGMP-dependent protein kinase (PKG) isoforms and guanylate cyclase (GC) in microneme secretion and host cell invasion (Brown et al., 2017; Brown and Sibley, 2018). This approach is very useful for establishing proteins essential to various aspects of parasite biology (e.g. the lytic cycle or bradyzoite conversion) and for identifying the role of specific parasite proteins in subverting the host immune response. This makes the AID system an exciting new approach to uncovering proteins that are of interest in the pursuit of novel vaccine targets for *T. gondii*.

The development and implementation of tools that allow for the genetic manipulation of apicomplexans, such as *T. gondii*, is very exciting and opens up new prospects for vaccine development. Through understanding gene and protein function in the context of parasite biology and host-parasite interactions, essential vaccine targets may be discovered. Genetically manipulated parasites could be used for the targeted development of more efficacious and safer live-attenuated strains, as well as be used to identify essential proteins that could be used in the production of a recombinant antigen-based killed vaccine.

## 8 Concluding remarks

A one health approach to develop a vaccination programme to tackle toxoplasmosis is an attractive and realistic prospect. Knowledge of disease epidemiology, parasite transmission routes and main risk groups has helped to target key host species and outcomes for a vaccine programme and these would be to prevent/reduce congenital disease in women and sheep; prevent/reduce *T. gondii* tissue cysts in food animal species and to prevent/reduce *T. gondii* oocyst shedding on cats.

*Toxoplasma gondii* is one of the most successful parasites worldwide and the same species can infect all warm blooded animals with the parasite invading and multiplying within host cells and differentiating from the fast multiplying tachyzoite stage to the persistent bradyzoite stage maintained within tissue cysts for the lifetime of the host. While there are some drugs available that can help to limit the fast multiplying tachyzoite stage of the parasite there are not drugs yet that are effective in eliminating parasites within tissue cysts. However, most animals and people that become infected with *T. gondii* develop a good immune response that will in the majority of cases protect them against disease going forward. This supports the strategy of using vaccination to tackle toxoplasmosis. Extensive studies to improve our understanding of host-parasite relationships in different host species, have emphasised the importance of CD8+ T cells and IFNу as key elements of a protective immune response which may explain why live vaccines, which enable appropriate processing and presentation of parasite antigens in the context of MHC class I antigens are so effective. Such live vaccines, comprised of attenuated *T. gondii* strains that do not persist in the host have been applied very successfully in veterinary medicine, resulting in one commercially available vaccine, but this would not be considered safe for use in people.

Exciting, recent advances in deciphering the *T. gondii* genome, identifying target vaccine antigens based on knowledge of key protective immune responses and the development of techniques to genetically manipulate the parasite have enabled new opportunities in vaccine development. Knowledge of new adjuvants and vaccine delivery systems are greatly improving the likelihood of developing safer *T. gondii* vaccine preparations that will stimulate the desired cell mediated immune responses in vivo. Improving our understanding of strain differences, in particular as regards virulence, will be important to ensure that vaccines will be effective against these pathogenic parasites.

A key factor in the successful development of a vaccine is the efficacy testing in a suitable model system and this is where a one health approach could really advance vaccine development. It is very important due to differences in host-parasite interactions to work where possible using relevant animal models. Currently it is difficult to compare the results of different vaccine experiments, even within the same host species, due to different protocols, routes of immunisation, challenge doses and parasite strains. However, it is possible to work together to devise standard vaccine efficacy challenge models in different target host species such as the cat, the sheep, the pig and work using MHC class I transgenic mice as a step towards a human vaccine. This would greatly advance progress towards a one health vaccination programme.

Toxoplasmosis is a serious disease with global impact, now recognised as one of the most important food borne diseases worldwide and a major cause of production loss in livestock. Vaccination is a strategy that can work to help tackle disease caused by the parasite and a one health approach has many advantages in this respect. One of the legacies from the conference held in Brazil in 2008, to celebrate 100 years since the discovery of *Toxoplasma gondii* is for researchers to come together from across the different disciplines and sectors and work collaboratively to make a difference and an achievable goal would be to develop a one health vaccine programme to tackle toxoplasmosis.
